# Carriage of methicillin-resistant *Staphylococcus pseudintermedius *in dogs--a longitudinal study

**DOI:** 10.1186/1746-6148-8-34

**Published:** 2012-03-23

**Authors:** Ulrika Windahl, Elin Reimegård, Bodil Ström Holst, Agneta Egenvall, Liselotte Fernström, Mona Fredriksson, Gunilla Trowald-Wigh, Ulrika Grönlund Andersson

**Affiliations:** 1 Department of Animal Health and Antimicrobial Strategies, National Veterinary Institute, SVA, SE-751 89 Uppsala, Sweden; 2 Södra djursjukhuset, Månskärsvägen 13, 141 75 Kungens kurva, Sweden; 3 Department of Clinical Sciences, Swedish University of Agricultural Sciences, SE-75007 Uppsala, Sweden; 4 Department of Biomedical Sciences and Veterinary Public Health, Swedish University of Agricultural Sciences, Box 7009, SE-750 07 Uppsala, Sweden

**Keywords:** MRSP, Methicillin resistant *Staphylococcus pseudintermedius*, Dog, Carriage, Bacterial infection

## Abstract

**Background:**

Methicillin-resistant *S. pseudintermedius *strains (MRSP) are reported with increasing frequency in bacterial cultures from dogs. The objectives of this study were to determine whether MRSP could be found in dogs several months after a clinically apparent infection and whether the length of carriage varied depending on systemic antimicrobial treatment, diagnosis at time of the first positive MRSP culture and the presence of skin disease or wounds. Thirty-one dogs previously diagnosed with a clinical infection were sampled repeatedly for a minimum of eight months or, with the exception of two dogs, until two consecutive negative results were obtained. Five specified locations were sampled, and the results were evaluated to determine future recommendations concerning sample strategies when screening for MRSP carriage. Information was collected from medical records and questionnaires to evaluate factors that may influence length of carriage.

**Results:**

The overall median length of MRSP carriage was 11 months (48 weeks). The presence of wounds and signs of dermatitis did not influence length of carriage. Systemic treatment for three weeks or longer with antimicrobial agents to which the bacterium was resistant was associated with prolonged carriage compared to dogs treated for a shorter period of time. Three of five dogs treated with an antimicrobial to which their MRSP-isolates were susceptible (tetracycline) were found to still be MRSP-positive when sampled after the end of treatment. Wound samples had the highest positive MRSP yield (81%) for the positive sample sites, compared to less than 70% for each of the other four sample sites. Cultures from the nostrils were less likely to detect MRSP carriage relative to the pharynx, perineum, wounds and the corner of the mouth.

**Conclusions:**

Dogs can carry MRSP for more than a year after a clinically apparent infection. Systemic antimicrobial treatment of infections with antimicrobial agents to which the MRSP-bacteria are resistant should be avoided when possible in dogs with possible or confirmed MRSP carriage or infection, since it may prolong time of MRSP carriage. Simultaneous sampling of pharynx, perineum, and the corner of the mouth as well as wounds when present is recommended when screening for MRSP. Cultures from nostrils were shown to be less likely to detect MRSP carriage.

## Background

*Staphylococcus pseudintermedius *is a part of the normal microbiota of dogs and an important opportunistic pathogen. It is most commonly associated with dermatologic infections, such as pyoderma, otitis externa and wound infections, but it is also capable of causing infections in other body tissues [1-4].

Since 2006 there has been a significant emergence of methicillin-resistant S. *pseudintermedius *(MRSP), mainly due to clonal spread [5-9]. Methicillin resistance is mediated by the *mec*A gene, which encodes the penicillin-binding protein (PBP) that has a low affinity for all ß-lactam antimicrobials [10].

Data on resistance in *S. pseudintermedius *in isolates from Swedish dogs referred to the National Veterinary Institute from veterinary clinics and hospitals has been reported since 1992 [11]. In 2006, the first 13 isolates of MRSP in Swedish dogs were reported. In 2007 and 2008, more than 180 MRSP isolates were confirmed [11]. This has necessitated evidence-based recommendations on infection control measures to prevent further spread in the dog population. One central issue is whether individual dogs, once found to be infected with MRSP, can become long-term carriers.

The objectives of this study were to determine whether MRSP can be found in dogs several months after a clinically apparent infection and whether length of carriage varies depending on systemic antimicrobial treatment, diagnosis at the time of the first positive MRSP culture and the presence of skin disease or wounds. Culture results from five sample sites were compared.

## Methods

### Dogs

The 31 dogs enrolled in the study had MRSP-positive cultures of clinical specimens (inclusion sample) referred from a total of eight veterinary clinics and hospitals to the SVA (National Veterinary Institute, Sweden) for diagnostic purposes between October 2007 and February 2009.

A copy of each dog's medical record was collected. In addition, a brief questionnaire was completed by the owner aided by the veterinarian at each sampling. The owners were asked if the dog had received any kind of treatment by other veterinarians before and during the study and if any signs of illness or dermatological changes had been noted since the last sample occasion. Data on breed, gender, age, presenting complaint (diagnosis), medical treatment and antimicrobial treatment from a year prior to the inclusion sample and during the entire study (sampling period) were compiled.

### Sample strategies

The dogs were sampled using bacterial swabs placed in Amies medium^® ^(Copan Italia S.p.A Brescia Italy). Samples were collected from four specified locations on all dogs; nostrils, pharynx, perineum and the corner of the mouth. Wounds were sampled when present. A separate swab was used for each location. Dogs in the study were to be sampled with intervals of 6-15 weeks until two negative results were obtained or for a minimum of eight months.

### Bacterial isolation, phenotypic identification and antimicrobial susceptibility testing

The samples were transported overnight and processed on the day of arrival at the laboratory. The swabs were placed in 10 mL tryptone soy enrichment broth (TSB) with 4% saline, 1% mannitol, 16 mg/L phenol, 50 mg/L aztreonam (MP Biomedicals) [12,13] modified by using 1 mg/L of cefoxitin (Sigma-Aldrich). After 48 hour incubation at 37°C, 10 μL of broth was plated onto two media: bovine blood-agar (National Veterinary Institute, Uppsala, Sweden) and mannitol salt agar with lithium chloride (Oxoid Ltd, Merck KGaA).

The plates were incubated aerobically at 37°C for 48 hours. Colonies were identified as suspected members of the *S. intermedius *species group (SIG) based on colony morphology, production of haemolytic toxins, coagulation of plasma, DNAse production and aerobic acid production from bromcresol-purple agar with 1% maltos and trehalose broth (SVA, Uppsala, Sweden).

All suspected SIG isolates were tested for susceptibility to oxacillin. The resistance breakpoint of ≥ 0.5 mg/L for oxacillin was used as an indicator for methicillin resistance. This breakpoint was recommended by Bemis and co-workers [14], and it has recently been approved by the CLSI subcommittee on Veterinary Antimicrobial Susceptibility Testing [15]. Isolates found to be resistant were examined further by polymerase chain reaction (PCR), as described below. MRSP isolates from the inclusion sample and from the last sample occasion were in addition tested for susceptibility to the following antimicrobial agents: penicillin, cephalothin, erythromycin, chloramphenicol, clindamycin, tetracycline, fusidic acid, gentamicin, kanamycin, ciprofloxacin and trimethoprim. Minimum inhibitory concentrations (MICs) of the antimicrobial agents were determined by broth microdilution, according to the recommendations of the Clinical and Laboratory Standards Institute (CLSI) [16] using VetMIC microdilution panels (National Veterinary Institute, Uppsala, Sweden). *S. aureus *ATCC 29213 served as quality control strain. The MIC breakpoints for classification of isolates as resistant were those recommended for *Staphylococcus *species in CLSI documents M100-S19 and M31-A3 [17,18]. For fusidic acid, the clinical breakpoint for *S. aureus *defined by the European Committee on Antimicrobial Susceptibility Testing (EUCAST) [19] was used.

### Molecular identification and PCR detection of *mec*A

DNA extraction was performed according to Capurro et al. [20]. Fragments corresponding to the 16S rRNA gene and the SIG specific thermonuclease gene were analysed by multiplex-PCR, as described previously by Baron et al. [21]. Detection of the *mec*A gene was performed in a single PCR [22]. As all isolates were from dogs, isolates positive for both the thermonuclease gene and the *mec*A gene were considered to be MRSP as previously described [5,7,23-25].

### Typing of strains

For 26 of the 27 dogs which were found to be MRSP-positive on one or more sample occasions following the inclusion sample, an isolate from the last positive sample occasion could be compared to either the inclusion sample (n = 21) or the first sample collected after inclusion (n = 5) by pulsed-field gel electrophoresis (PFGE). The dog whose inclusion isolate was lost to follow up was found to be negative on the following sample occasion, within 26 weeks (6 months) from the inclusion sample. *Sma*I macrorestriction patterns were obtained using the standardised HARMONY protocol [26], but with the pulse switch times as described by Perreten et al. [7]. For comparison of *Sma*I patterns, cluster analysis was performed by comparing gel images using the BioNumerics software (version 4.5; Applied Maths, Sint-Martens-Latem, Belgium).

### Statistical analyses

The outcome of interest, MRSP-negativity, was in this study defined as the cultures from all sample sites being MRSP-negative on two consecutive sample occasions. Length of MRSP-carriage was defined as time from the inclusion sample until the first of two consecutive negative sample occasions, with the exception of two dogs that were found to be negative on only one sample occasion, after which they left the study. These two dogs were therefore regarded as MRSP-negative on that last sample occasion.

The Kaplan-Meier (product limit) method was used to create probability curves for length of MRSP carriage as well as the median time (i.e., when 50% of the dogs were found to be MRSP-negative). This was done crudely, as well as by gender, age, diagnosis at time of inclusion sample, presence of dermatitis or wounds during the study, and relative to the time during which the dogs had received systemic treatment with antimicrobial agents to which the cultured MRSP were resistant. All such antimicrobial treatment received within three months prior to and during the study period was included in the analysis. For comparison, the median time of carriage was in addition calculated with the last positive sample occasion for each dog as the end of MRSP carriage.

In the analysis, gender was categorised as male or female with no distinction between intact and spayed or neutered dogs. Six years was the mean and median age of the population and for the age variable, dogs ≤ 6 years (n = 17) was contrasted to older dogs (n = 14). The variable "length of antibiotic treatment" was categorised in two groups. Length of treatment ≥ 21 days with antimicrobials to which the cultured MRSP bacteria were resistant was contrasted to shorter treatment periods. Diagnosis at time of inclusion sample was categorised into three diagnostic categories: dermatitis (n = 11), surgical procedures (n = 13) or infection/trauma (n = 7), according to the clinical diagnosis made by the veterinarians sampling the dogs. At the stratification, the log-rank test was used to test whether probability curves differed and *p *< 0.05 was considered significant. The median number of positive sample sites (wounds excluded) found in each dog per positive sample occasion relative to presence of wounds and dermatitis respectively was compared. All statistical analyses above were performed using SAS statistical software (SAS version 9.1, SAS Institute Inc., Cary NC). The nonparametric two-sample Mann-Whitney Test (Minitab) was used to compare the median number of positive sample sites found in each dog per positive sample occasion relative to presence of wounds and dermatitis respectively.

### Ethics

The study was approved by the Swedish Board of Agriculture and the Ethical Committee on Animal Experiments in Uppsala. Written consent from each dog owner was collected by the veterinarian on each sample occasion using a specific consent form approved by the Ethical committee.

## Results

Twenty-four breeds were represented, only three of which were observed in more than one individual dog. Four dogs were of mixed breed. Mean and median age at the time of the inclusion sample was six years (range 1-12 years). Sixteen of the 31 sampled dogs were intact females, nine were intact males, three were spayed females, and three were neutered males.

Thirteen dogs were diagnosed as having post-operative infections at the time of the inclusion sample. Eleven dogs were diagnosed with dermatitis at the time of the inclusion sample. The remaining seven dogs were treated for traumatic injuries or secondary infection.

Six of the eleven dogs with dermatitis at the time of the inclusion sample had recurrence of skin disease later during the study. Signs of dermatitis were present on ten sample occasions, three of which yielded negative results from all sample sites. Only one of the 31 dogs (dog No. 20, Additional file 1: Table S1) showed other clinical signs of infection during the sampling period. That dog had an oronasal fistula and a yellow, thick nasal discharge was noted by the owner daily during the entire study period.

All dogs received systemic treatment with antimicrobial agents to which the cultured MRSP were resistant prior to the first sample occasion in the study. Median length of treatment was 24 days. No dogs received further treatment with such antimicrobials during the study, and in approximately two thirds (n = 20) of the dogs, the treatment was ended prior to, or at the time of, the inclusion sample. Five dogs received systemic treatment with tetracycline, an antimicrobial agent to which the cultured MRSP isolates were susceptible. The treatment was ended prior to the first sample occasion in four of these dogs. The fifth dog (dog No. 31, Additional file 1: Table S1) was the only dog in the study receiving any antimicrobial treatment after the first sample occasion of the study. The median length of treatment with tetracycline was 30 days (range 20-60 days).

**Figure 1:**
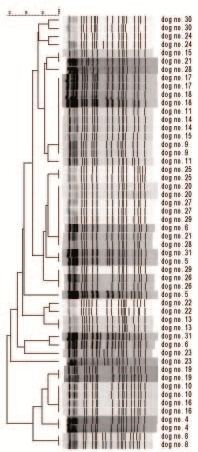
**Dendogram of the 26 pairs of isolates from 26 dogs compared by pulsed-field gel electrophoresis**.

The *Sma*I restrictions profiles showed 85% or more similarity between the two isolates compared by PFGE from each dog (Figure 1). With the exception of isolates found in dog number 17 and 20, the MRSP-isolates tested for antimicrobial susceptibility showed similar antibiograms, being susceptible only to fusidic acid and tetracycline. The isolates with diverging results were found to be resistant to tetracycline.

**Figure 2:**
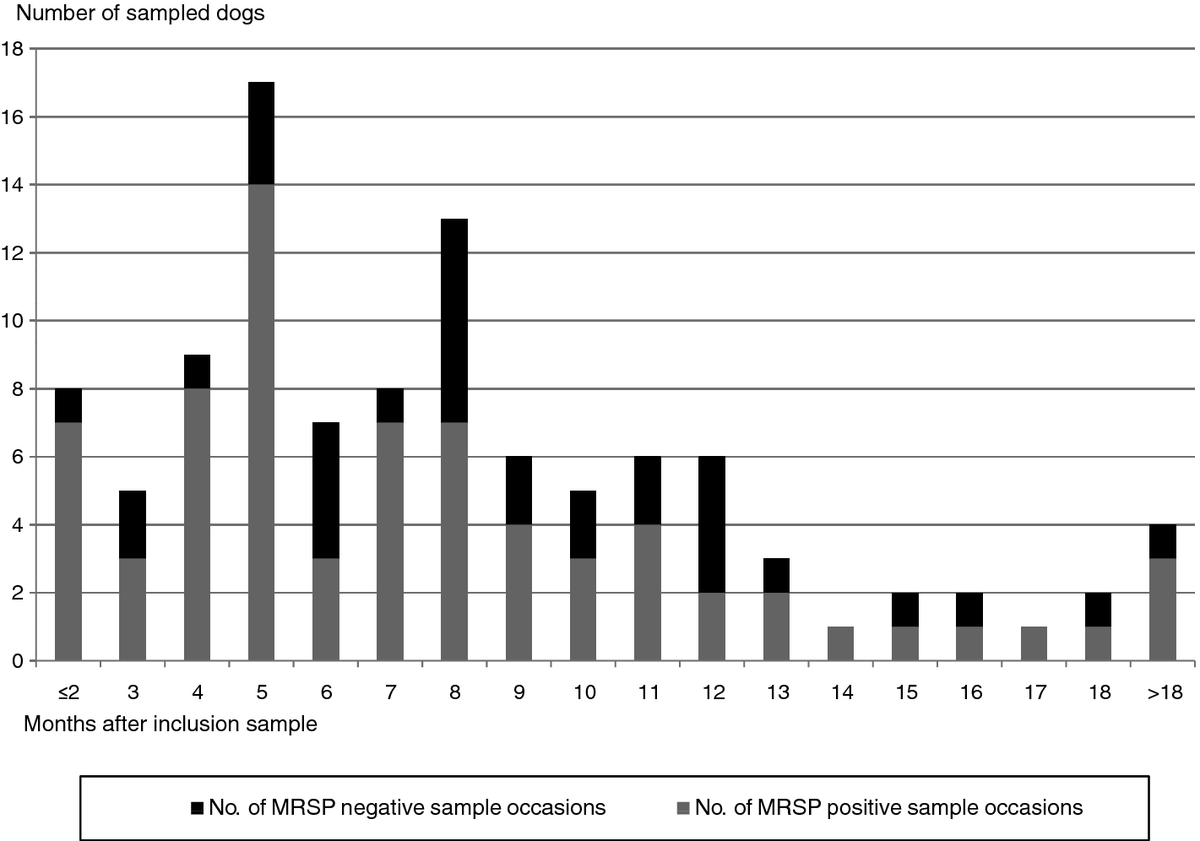
**Overview of sampled dogs over months**.

Twenty-nine out of the 31 dogs were sampled according to plan regarding length of follow-up. Two dogs were only available for sampling for 5.5 and 6 months, respectively. All samples were collected according to the planned minimum interval and 60% within the planned 3.5 month maximum interval. The median study period was 10 months (range 4.5-19), and the median number of sample occasions per dog was 3 (range 2-6). The median time between sample occasions was 2.5 months (Additional file 1: Table S1, Figure 2).

Five dogs remained MRSP-positive for more than 14 months (61 weeks). Nine dogs were sampled for 5-12 months (22-52 weeks) and remained positive. One of these dogs had a negative result on one sample occasion followed by positive results. Fifteen dogs were found to be negative within 12 months (52 weeks) and they remained negative 3.5 to 7.5 months later (15-33 weeks). Two dogs left the study at five and ten months, respectively, with negative results from all sample sites at the final sample occasion (Additional file 1: Table S1).

**Figure 3:**
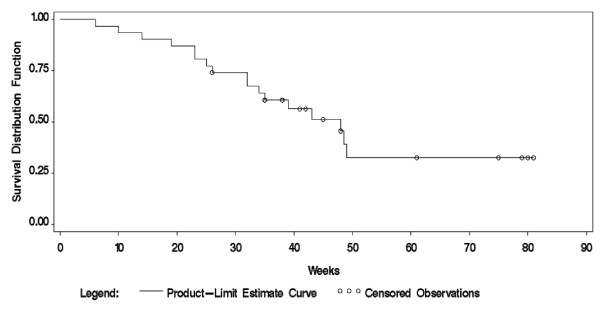
**Proportion of dogs that were MRSP-negative in relation to time from inclusion sample**. The proportion of dogs found to be MRSP-negative (y-axis) in relation to time in weeks from inclusion sample (x-axis). MRSP negativity was defined as the cultures from all sample sites being MRSP-negative on two consecutive sample occasions; however, two dogs with one negative sample occasion each are included. Out of a total of 31 dogs, 17 became MRSP-negative. The last positive sample occasion for each of the 14 dogs that remained positive when leaving the study are shown as censored observations.

The overall median length of MRSP carriage was 11 months (48 weeks) (Figure 3). When the last positive sample occasion was used as the end of MRSP carriage the median time of carriage was 45 weeks. No significant differences in length of MRSP carriage appeared either between the three diagnostic categories (*p *= 0.31) or in the dogs with dermatitis on the following sample occasions in the study compared to dogs with no visible signs of skin disorder (*p *= 0.78). Possible influence of breed could not be evaluated from the material due to the wide variety of breeds being represented in the relatively small number of dogs. There was no significant difference in the length of carriage between the two age groups: ≤ 6 years versus > 6 years (*p *= 0.43).

**Figure 4:**
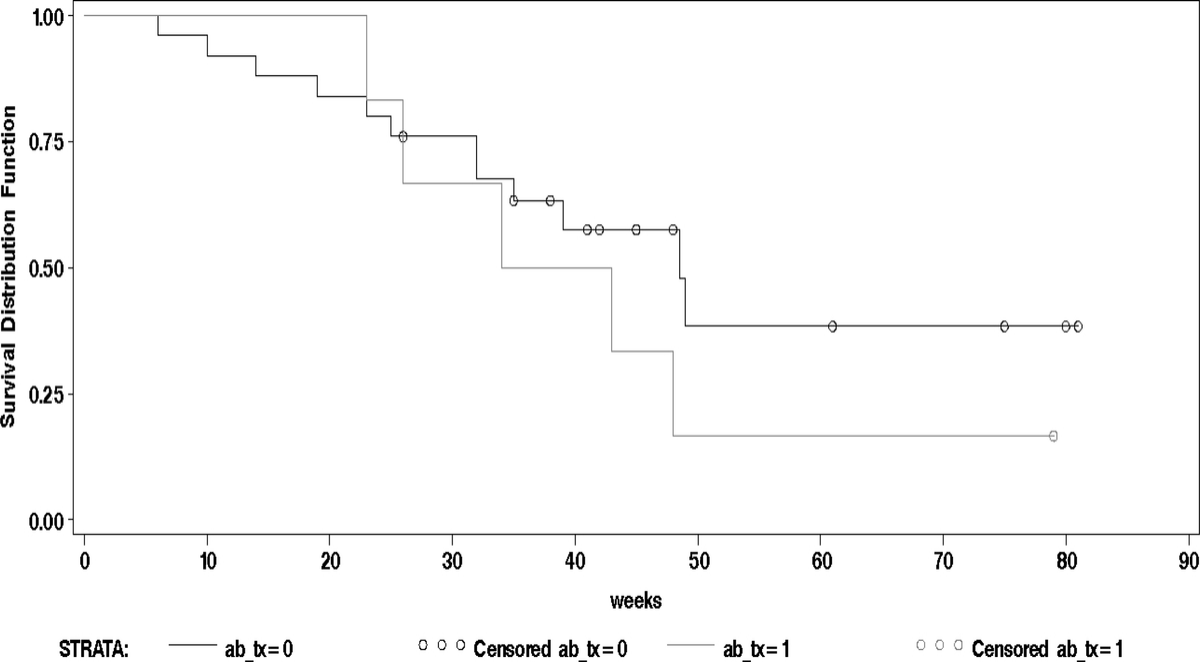
**Length of MRSP carriage from inclusion sample related to length of antimicrobial treatment**. The proportion of dogs found to be MRSP-negative (y-axis) in relation to time in weeks from the inclusion sample (x-axis) by length of systemic treatment with antimicrobial agents to which the MRSP isolates were resistant. Dogs (n = 19) treated for three weeks (≥ 21 days) are contrasted to dogs with shorter treatment periods (n = 12).

A statistically significant difference in length of MRSP carriage was found between dogs treated with antimicrobials to which the cultured MRSP bacteria were resistant for a period of three weeks (21 days) or longer (n = 19) and dogs treated for a shorter period (n = 12) (*p *= 0.01). Median length of carriage was 11.3 months (48.5 weeks) for the "long-term treatment" group and 5.7 months (24.5 weeks) for the group treated for a shorter period (Figure 4).

Two of the five dogs receiving systemic treatment with an antimicrobial agent to which their cultured MRSP isolates were susceptible (tetracycline) were found to be MRSP-negative on the following sample occasion. The first dog (dog no 1) was treated for three weeks and was found to be negative on the very first sample occasion in the study, 8 months (34 weeks) after the inclusion sample. The second dog (dog No. 31) was treated for 4 weeks in between being sampled at 8.5 and 10 months (37 and 43 weeks) after the inclusion sample. The dog was unavailable for further sampling. The remaining three dogs were found to be MRSP-positive on the sample occasions following end of treatment. Length of proven MRSP carriage was nine months (39 weeks) following 8.5 weeks of treatment (dog No. 5) and approximately five to six months (23 and 26 weeks) following 4.3 weeks of treatment (dogs No. 30 and 7). Median length of carriage of MRSP for the five dogs that received treatment with tetracycline was 34 weeks, compared to 49 weeks in non-treated dogs (*p *= 0.06).

MRSP could be isolated from all sample sites. On 9 of the in total 73 positive sample occasions, positive cultures were found from all four sample sites. On 21 occasions MRSP was found from only one of the sampled sites. The nose was the only sample site that never yielded a positive culture when all other sites were negative. MRSP was isolated from the nostrils in only 38% of the 73 positive sample occasions. From the corner of the mouth, perineum and pharynx MRSP was isolated in 58, 63, and 67%, respectively, of the positive sample occasions.

Nonpurulent wounds were present in 11 dogs on a total of 20 sampling occasions, excluding the inclusion samples. Ten dogs had one or several wounds when first sampled, and five of these still had wounds on the following sampling. Two of these dogs were found to be negative on both of these sample occasions. MRSP was isolated from wounds in 13 of the 16 positive samplings of dogs with wounds present (81%). All dogs with a positive MRSP wound culture also had simultaneous positive cultures from one or several other sampling sites.

The presence of nonpurulent wounds did not significantly increase the length of MRSP carriage (*p *= 0.94). It did however significantly increase the number of positive sample sites (*p *= 0.003). The median number of positive sample sites per positive sample occasion, excluding wounds, in each dog was 2.66 when wounds were present (9 dogs) and 2.0 when there were no wounds (18 dogs).

No significant association could be found between the median number of positive sample sites per sample occasion and presence vs. absence of dermatitis during the study (2.00 and 2.33 respectively, *p *> 0.9).

## Discussion

This study shows that dogs can carry MRSP for several months. Nineteen dogs (61%) were found to be MRSP-positive for at least eight months. Five of these were followed for more than 14 months (60 weeks) and remained positive. The exact length of carriage is not possible to determine because the period in between samplings varied depending on practicalities, such as when the dogs were available for sampling. However, it is worth mentioning that the median time of carriage was very similar regardless if the first negative sample (48 weeks) or the last positive sample (45 weeks) was used as the end of carriage. It is also unknown if, and for how long, the dogs were already carrying MRSP before the inclusion sample.

Reinfection of dogs during the study cannot be ruled out. Contact with other MRSP colonized dogs or humans might have served as a source of reinfection, as well as contaminated objects in the household [24]. Re-infection might also have occurred during visits to the animal clinics and - hospitals, despite strict hygiene measures being enforced in connection with all aspects of handling and caring for the dogs after the initial MRSP finding (i.e. the inclusion sample) was reported.

Emergence of MRSP has led to several investigations of the phylogenetic background, diversity and clonal distribution using different molecular and phenotypical approaches e.g. [5-8,27]. Conclusive criteria on interpretation of available typing methods for MRSP in relation to strain persistence are currently lacking. Kadlec et al. [28] examined genetic relatedness of isolates obtained from the same dogs during a time period of up to approximately ten months, and found some *Sma*I fragment patterns to be indistinguishable, and others with distinct differences. Most studies on MRSP are however one-point prevalence studies, and to what extent changes occur over time within a carrier needs to be elucidated. Further studies investigating MRSP isolates from the same patient over longer periods of time are important for increased knowledge and development of more definitive tools of investigation.

Of the fifteen dogs that were sampled until two consecutive negative results were achieved, twelve were found to be MRSP-negative within nine months (39 weeks) from the time of the inclusion sample, the remaining three within twelve months. Four of these dogs were found to be MRSP-negative on their very first sample occasion. According to the four dogs' medical charts, the inclusion sample was the first bacteriological sampling made in connection with the relevant diagnosis. It can therefore not be ruled out that the finding of MRSP in the inclusion sample of these dogs was a transient contamination. It is possible that all or some of the 14 dogs leaving the study with a positive result would also have become MRSP-negative if sampled for longer.

Data published on carriage of MRSP in dogs are limited, and guidelines regarding the possibility of declaring a dog as no longer carrying MRSP are lacking. In the present study, two consecutive negative sample occasions were used to define the end of MRSP carriage to decrease the risk of a false negative laboratory result. Possible causes for a false negative culture result include failure to identify and further culture potential MRSP colonies at the laboratory. Because the samples in the study did not originate from a clinically infected site, there was also an increased risk of not being able to detect the bacteria of interest due to overgrowth of other bacteria and fungi, despite the use of selective enrichment broth and selective agar. In the present study, cefoxitin was included in the enrichment broth. Recent studies have demonstrated that the use of cefoxitin disk diffusion susceptibility testing produces false negative results when screening for methicillin resistance in veterinary isolates of *S. pseudintermedius *[14,15]. However, this does not mean that cefoxitin cannot be used in the lower concentration 1 mg/L in the enrichment broth, as in the present study. Only one of the 31 dogs had a negative culture result from all four sites between positive sample results (sample occasion 2 of 4).

In this study, a significant difference in the duration of MRSP carriage was found between dogs receiving general treatment with antimicrobial agents to which the bacteria were resistant for three weeks or longer and dogs treated for a shorter period with such antimicrobials. Although, to the best of our knowledge, published studies on use of antimicrobial drugs selecting for MRSP in dogs are lacking, logical hypotheses and biological data, including those from human medicine regarding MRSA (methicillin-resistant *S. aureus*) carriage and infection, support our finding. The normal skin flora is part of the skin's defence mechanisms. The normal bacterial skin flora occupy microbial niches and inhibit colonisation by invading organisms [2], and it has been shown that antimicrobial treatment can enable the survival and colonisation of pathogenic bacteria resistant to the antimicrobial agent in question by suppressing part of this flora [29,30]. Dogs receiving systemic treatment with antimicrobials to which the bacterium is resistant may therefore have a higher risk of carrying, spreading, and developing clinical infections with MRSP. The risk for facilitating bacterial growth and thereby prolonging clinically apparent infections by prescribing antimicrobials should also not be overlooked.

The relatively small sample size (five dogs) combined with the variation in sampling strategies limits the possibility of evaluating the effect of treatment with an antimicrobial agent to which the MRSP isolates were susceptible on the length of carriage of the bacteria. It is however noteworthy that three of the dogs were still found to be MRSP-positive for several months after the four to nine week-long treatment had ended. This suggests that MRSP established itself as a part of the normal microbiota, which would prevent total eradication.

No statistically significant difference in the length of MRSP carriage was found either between the diagnostic categories or with the presence (or absence) of nonpurulent wounds or signs of dermatitis during the study. In human medicine, the risk of becoming colonised and carrying MRSA on the skin, (and thereby the risk of spreading the bacteria to other individuals) has been shown to increase with such skin changes [31,32]. Lloyd and co-workers [33] found that dogs with superficial bacterial folliculitis had larger populations of *S. intermedius *(canine *S. intermedius *are now considered to be *S. pseudintermedius*) on their nonlesional skin compared to clinically normal dogs, and two studies found increased populations of the bacteria on mucosal surfaces in dogs with pyoderma [34,35]. Although our data do not support an association between the presence of skin lesions and the length of MRSP carriage the sample size in each of the groups in our study was relatively small, resulting in a corresponding limitation in statistical power. Further studies with larger sample sizes might provide more information regarding factors associated with the length of detectable MRSP carriage.

Nonpurulent wound samples (inclusion samples excluded) had the highest positive MRSP yield (81%) of the positive sample occasions, compared to less than 70% for each of the four other sample sites (i.e., the nostrils, pharynx, perineum and the corner of the mouth). The nostrils was found to be the most difficult site to sample correctly and had the lowest positive yield (38%). Because almost 20% of the wound samples were negative, despite the bacteria being found in cultures from other sites that were sampled simultaneously, we conclude that a negative nonpurulent wound culture should not be used as a definitive criterion for a dog being MRSP-negative. In a previous study where colonisation frequency with MRSP was compared in various sample sites: dorsal skull, the buccal and gingival mucosa, the distal mucosa of the nares, the ventromedial inguinal fold, and the external anus, no site was found to be more likely to carry methicillin-resistant *staphylococci *[36]. The reason for discrepancy between this study and our results is not known.

## Conclusions

In conclusion, our study shows that dogs can carry MRSP for several months without clinical signs. The presence of wounds or signs of dermatitis did not influence length of carriage.

Systemic treatment with antimicrobial agents to which the bacterium was resistant for three weeks (21 days) or longer was associated with prolonged carriage compared to dogs treated for a shorter period. In addition, three of five dogs treated with an antimicrobial to which their MRSP-isolates were susceptible (tetracycline) were found to still be MRSP-positive when sampled after the end of treatment. We therefore recommend that systemic antimicrobial treatment in dogs with possible or confirmed MRSP carriage or infection should be avoided when possible.

Based on our results, simultaneous sampling of several body sites when screening clinically healthy dogs for MRSP is recommended. Our results also suggest that the nostrils are not a priority when screening dogs for MRSP. Furthermore, whenever the culture from the nostrils was MRSP-positive, MRSP could also be found in one or more of the other sites.

## Abbreviations

MRSP: Methicillin-resistant *S. pseudintermedius; *SVARM: Swedish veterinary antimicrobial resistance monitoring; SVA: National Veterinary Institute Sweden (Statens veterinärmedicinska anstalt); PBP: Penicillin-binding protein; PCR: Polymerase chain reaction; MICs: Minimum inhibitory concentrations; CLSI: Clinical and Laboratory Standards Institute; EUCAST: The European committee on antimicrobial susceptibility testing; PFGE: Pulsed-field gel electrophoresis; MRSA: Methicillin-resistant *S. aureus*.

## Competing interests

The authors declare that they have no competing interests.

## Authors' contributions

UW designed and coordinated the study, participated in the bacterial analyses and drafted the manuscript. AE and BSH performed the statistical analyses and drafted the manuscript. LF and MF carried out the bacterial analyses, including the PCR analyses. UGA, ER and GTW participated in the design of the study and of the manuscript. All authors read and approved the final manuscript.

## Supplementary Material

Additional file 1 **Table S1**. Information on dogs included in the study. Click here for file
