# Supplementary material for: Carriage of methicillin-resistant Staphylococcus pseudintermedius in dogs--a longitudinal study
Source: BMC Vet Res. 2012 Mar 23;8:34. doi: 10.1186/1746-6148-8-34 (PMC3325892; doi:10.1186/1746-6148-8-34)
Supplement: Additional file 1 — Table S1. Information on dogs included in the study. [file 1746-6148-8-34-S1.PDF]

		Length of antimicrobial treatment (days) to which the isolates were:			Time of carriage	No. of weeks from inclusion sample (N = Negative culture, P = positive culture) D = Dermatitis present, W = Wound present					
Dog No.	Diagnosis at time of inclusion	Susceptible (teracycline)	Resistant	Antimicrobials used to which the isolates were resistant	Weeks	Sample occasion 1	Sample occasion 2	Sample occasion 3	Sample occasion 4	Sample occasion 5	Sample occasion 6
1	Dermatitis	20	30	cephalexin	34	34 (N) D	51 (N)	.	.	.	.
2	Surgery		20	amoxicillin	6	6 (N) W	27 (N) W	.	.	.	.
3	Surgery		14	amoxicillin	10	10 (N) W	20 (N) W	.	.	.	.
4	Surgery		30	amoxicillin, cephalexin	39	22 (P)	39 (N)	67 (N)	.	.	.
5	Dermatitis	60	20	enrofloxacin	48	32 (P) D	48 (N)	77 (N)	.	.	.
6	Surgery		40	amoxicillin, enrofloxacin	48.5	28.5 (P)	48.5 (N)	82 (N)	.	.	.
7	Surgery	30	17	amoxicillin	26	9 (P)	26 (N)	54 (N)	.	.	.
8	Surgery		7	enrofloxacin	19	6 (P)	19 (N)	39 (N)	.	.	.
9	Surgery		35	ampicillin, enrofloxacin	32	19 (P)	32 (N)	47 (N)	.	.	.
10	Surgery		35	cephalexin	32	8 (P) W	32 (N)	50 (N)	.	.	.
11	Infection, trauma		9	amoxicillin, cephalexin	49	18 (P)	32 (P)	49 (N)	64 (N)	.	.
12	Infection, trauma		3	enrofloxacin	14	14 (N)	20 (N)	.	.	.	.
13	Infection, trauma		14	cephalexin	23	17 (P)	23 (N)	33 (N)	.	.	.
14	Dermatitis		8	amoxicillin, enrofloxacin	35	19 (P)	27 (P)	35 (N)	43 (N)	.	.
15	Dermatitis		84	cephalexin	25	15 (P)	25 (N)	35 (N)	.	.	.
16	Infection, trauma		21	amoxicillin, cephalexin	80	5 (P)	21 (P)	50 (P)	69 (P)	80 (P)	.
17	Surgery		42	amoxicillin, enrofloxacin	79	11 (P) W	26 (P)	56 (P)	70 (P)	79 (P)	.
18	Surgery		30	amoxicillin, clindamycin	81	15 (P) W	22 (P)	32 (P)	45 (P)	81 (P) W	
19	Infection, trauma		28	amoxicillin, enrofloxacin, gentamicin	61	7 (P)	17 (P)	35 (P)	43 (P)	50 (P)	61 (P)
20	Infection, trauma		30	cephalexin, enrofloxacin	75	10 (P)	18 (P)	47 (P)	55 (P)	65 (P)	75 (P)
21	Dermatitis		26	clindamycin	35	14 (P)	21 (P)	28 (P)	35 (P)	.	.
22	Infection, trauma		15	enrofloxacin	38	17 (P) W	22 (P) W	32 (P) W	38 (P)	.	.
23	Dermatitis		60	cephalexin	48	12 (P) D,W	39 (P) D,W	48 (P) D	.	.	.
24	Surgery		30	enrofloxacin	38	15 (P)	30 (P)	38 (P)	.	.	.
25	Dermatitis		31	amoxicillin	35	15 (P)	21 (P)	27 (P)	35 (P)	.	.
26	Dermatitis		21	cephalexin, enrofloxacin	42	21 (P) D	35 (P)	42 (P)	.	.	.
27	Surgery		24	cephalexin	41	21 (P) W	30 (P) W	41 (P) W	.	.	.
28	Dermatitis		180	cephalexin, enrofloxacin	45	21 (P) W	45 (P) W	.	.	.	.
29	Dermatitis		17	amoxicillin, cephalexin, clindamycin	26	5 (P)	13 (N)	20 (P) D	26 (P)	.	.
30	Surgery	30	7	cephalexin	23	8 (P) W	23 (N)	.	.	.	.
31	Dermatitis	30	21	clindamycin	43	25 (P) D,W	37 (P) D	43 (N) D	.	.	.
